# Daily PM_2.5_ concentration estimates by county, ZIP code, and census tract in 11 western states 2008–2018

**DOI:** 10.1038/s41597-021-00891-1

**Published:** 2021-04-19

**Authors:** Colleen E. Reid, Ellen M. Considine, Melissa M. Maestas, Gina Li

**Affiliations:** 1 Geography Department, Campus Box 260, University of Colorado Boulder, Boulder, CO 80309 USA; 2 Earth Lab, 4001 Discovery Drive Suite S348 – UCB 611, University of Colorado Boulder, Boulder, CO 80309 USA; 3 Institute of Behavioral Sciences, 483 UCB, University of Colorado Boulder, Boulder, CO 80309 USA; 4 Applied Mathematics Department, Engineering Center, ECOT 225, 526 UCB, University of Colorado Boulder, Boulder, CO 80309 USA

**Keywords:** Atmospheric chemistry, Natural hazards

## Abstract

We created daily concentration estimates for fine particulate matter (PM_2.5_) at the centroids of each county, ZIP code, and census tract across the western US, from 2008–2018. These estimates are predictions from ensemble machine learning models trained on 24-hour PM_2.5_ measurements from monitoring station data across 11 states in the western US. Predictor variables were derived from satellite, land cover, chemical transport model (just for the 2008–2016 model), and meteorological data. Ten-fold spatial and random CV R^2^ were 0.66 and 0.73, respectively, for the 2008–2016 model and 0.58 and 0.72, respectively for the 2008–2018 model. Comparing areal predictions to nearby monitored observations demonstrated overall R^2^ of 0.70 for the 2008–2016 model and 0.58 for the 2008–2018 model, but we observed higher R^2^ (>0.80) in many urban areas. These data can be used to understand spatiotemporal patterns of, exposures to, and health impacts of PM_2.5_ in the western US, where PM_2.5_ levels have been heavily impacted by wildfire smoke over this time period.

## Abstract (summary)

**Table:** 

Measurement(s)	fine respirable suspended particulate matter
Technology Type(s)	machine learning
Factor Type(s)	elevation • land cover • vehicle emissions • seasonality • spatiotemporal variation
Sample Characteristic - Environment	air pollution
Sample Characteristic - Location	State of Arizona • State of California • State of Colorado • State of Idaho • State of Montana • State of Nevada • State of New Mexico • State of Oregon • State of Utah • State of Washington • State of Wyoming

Machine-accessible metadata file describing the reported data: 10.6084/m9.figshare.14161856

## Background & Summary

Fine particulate matter (often referred to as PM_2.5_, meaning particulate matter (PM) which is 2.5 microns in aerodynamic diameter or smaller) air pollution is increasingly associated with numerous adverse health outcomes including, but not limited to, mortality^1^, respiratory and cardiovascular morbidity^2,3^, negative birth outcomes^4^, and lung cancer^5^. Although PM_2.5_ concentrations have been declining in many parts of the United States due to policies to limit emissions of air pollutants^6^, PM_2.5_ levels have been increasing in parts of the western US^7^. This increase has been shown to be associated with wildfire smoke^7,8^, which can cause PM_2.5_ concentrations that are several times higher than the Environmental Protection Agency’s (EPA’s) daily PM_2.5_ National Ambient Air Quality Standard (NAAQS) in areas downwind of the wildfires for several days at a time^9^.

Estimates of PM_2.5_ concentrations for health studies have traditionally been derived from data from stationary air quality monitors placed in and around populated areas for regulatory purposes. In the US, the EPA’s Federal Reference Method (FRM) PM_2.5_ monitors often only measure every third or sixth day and most counties do not contain a regulatory air pollution monitor^10^. There is therefore not enough temporal and spatial coverage from FRM monitors to obtain a good estimate of the air pollution exposures where every person lives.

To improve population exposure assessment of PM_2.5_, researchers have increasingly been using methods to estimate PM_2.5_ exposures in the temporal and spatial gaps between regulatory monitoring data using data from satellites (such as aerosol optical depth (AOD) or polygons of smoke plumes or air pollution models^11,12^) over the past two decades. Each of these data sources has its own benefits and limitations, and researchers are increasingly statistically “blending” information from a combination of data sources to better estimate PM_2.5_ in space and time. Various methods of blending have been used including spatiotemporal regression kriging^13^, geographically-weighted regression^14^, and machine learning methods^15–19^.

Machine learning methods for estimating PM_2.5_ concentrations often train large auxiliary datasets, often including satellite AOD, meteorological data, chemical transport model output, and land cover and land use data to provide optimal estimates of PM_2.5_ where people breathe. These models have been implemented in various locations around the world at city, regional, and national scales^20^. Some epidemiological questions can only be addressed in longitudinal studies with large sample sizes. Exposure models with large spatial and temporal domains will help enable such studies. Within the US, Di *et al*. (2016; 2019)^16,17^, Hu *et al*. (2017)^18^ and Park *et al*. (2020)^19^ have separately used machine learning algorithms to create fine-resolution daily PM_2.5_ estimates for the continental US. These models, however, have performed poorly in the western US^16,18^ and particularly the mountain west^17^ compared to the rest of the country. Given the increasing trends in PM_2.5_ concentrations in parts of the western US^7,8^ and the importance of wildfires as a source of PM_2.5_ there, it is important to have a model that is tailored to this region to capture the variability in PM_2.5_ concentrations in space and time in this region.

**Fig. 1:**
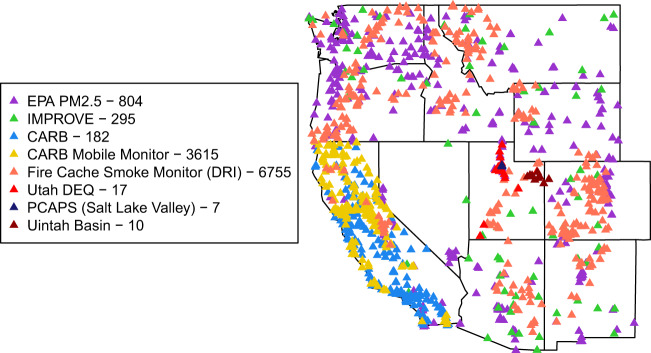
PM_2.5_ Monitoring Locations by Source of Monitoring Data. This map shows the locations of all PM_2.5_ monitors used to train the machine learning models that created the daily PM_2.5_ surfaces.

The dataset we describe here improves upon previous daily estimates of PM_2.5_ concentrations from machine learning models in the following ways: (1) use of a more extensive monitoring station network that captures more spatial locations (Fig. 1), some of which are closer to wildfires, a key driver of PM_2.5_ in the western US, (2) retaining high PM_2.5_ monitoring observations and estimating for years with many wildfires to train on, thus likely allowing our models to better predict the high PM_2.5_ values that occur during wildfire episodes, (3) employing robust spatial cross-validation techniques and 10% of monitoring locations that were completely left out of training on which to evaluate our models, (4) creating a long time series of daily PM_2.5_ concentration estimates at three geographic levels (county, ZIP code, and census tract) that can be applied in environmental health studies and (5) making the data available in a public repository. The data are available as daily PM_2.5_ concentration estimates at census tract, ZIP code, and county scales with the aim that they be used by researchers to understand the societal impacts of air pollution exposure in the western US, where wildfires are a significant contributor to PM_2.5_ concentrations.

## Methods

**Fig. 2:**
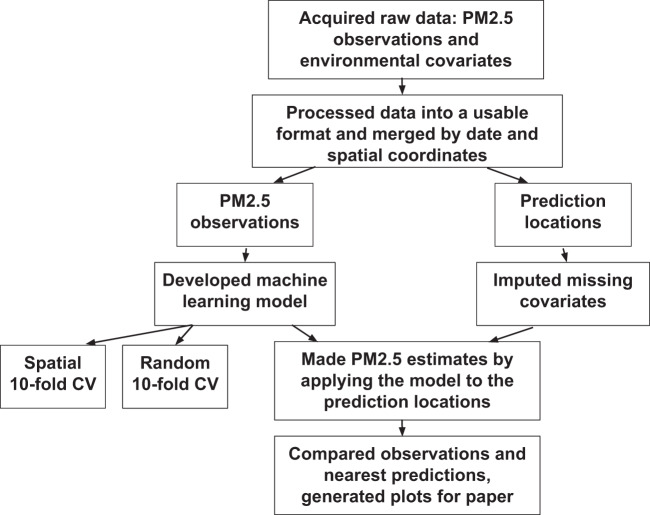
Flowchart diagram of methods to create daily PM_2.5_ concentration estimates for ZIP codes, census tracts, and counties throughout the western US from 2008–2018.

An overview of our methods is shown in Fig. 2.

### Study area

Our study area includes 11 western US states: Arizona, California, Colorado, Idaho, Montana, Nevada, New Mexico, Oregon, Utah, Washington, and Wyoming (Fig. 1). Our temporal domain includes all days between January 1, 2008 and December 31, 2018.

### PM_2.5_ Measurements

Regulatory monitoring for PM_2.5_ is done by the US EPA and their data is freely available on their website. In the western US, however, these monitoring sites do not represent all regions very well. We did an extensive search for as many PM_2.5_ monitoring data within our spatial and temporal study area as we could find, including from state governments and university researchers. We downloaded PM_2.5_ data (including parameter codes 88101, 88500, 88502, 81104) from all of the following sources: EPA Air Now data^21^, EPA IMPROVE Network^22^, California Air Resources Board (stationary and mobile monitoring networks)^23^, Federal Land Manager Environmental Database^24^, Fire Cache Smoke Monitor Archive^25^, Utah State University^26^, Utah Department of Environmental Quality^27^, and the University of Utah^28^. These data provided a more comprehensive set of locations and time points of PM_2.5_ measurement throughout the western US than are included in most PM_2.5_ modelling studies. The EPA IMPROVE monitors capture air quality information in more rural areas^22^. PM_2.5_ data in the Fire Cache Smoke Monitor Archive^25^ includes U.S. Forest Service monitors that were deployed to capture air quality impacts during wildfire events – the high number of geographic locations for these monitors in Fig. 1 (Fire Cache Smoke Monitor (DRI)) is because these are mobile monitors that are placed for each wildfire event and our data is showing the number of geographic locations for a given dataset rather than unique monitors. Any data that was repeated from multiple sources were removed. Also, all observations above 850 µg/m^3^ (N = 52 or 0.002% of our training observations), many of which were above 1000 µg/m^3^, were removed due to concern about the validity of these observations. This yielded a total of 1,589,462 daily PM_2.5_ observations, which represent 7,753 locations and 4,006 days. Descriptive statistics for the PM_2.5_ observations are in Supplementary Table 1.

### Predictor variables

The predictor variables for the machine learning ensemble are listed in Online-only Table 1 and described in more detail below.

Satellite AOD is a measure of particle loading in the atmosphere from the ground to the satellite. We obtained daily estimates of AOD from the MODIS Terra and Aqua combined Multi-angle Implementation of Atmospheric Correction (MAIAC) dataset^29^. This is the finest resolution (1 km) AOD dataset currently available and was available for our whole time period and spatial domain. After downloading each Hierarchical Data Format (HDF) file from the online repository, we calculated the average daily AOD values at each location and took the value from the nearest neighbour at each PM_2.5_ monitoring location. MAIAC AOD has been shown to better predict PM_2.5_ than coarser resolution AOD^30^ and has been used in many studies in various geographic regions in blended models to predict daily PM_2.5_^31–33^.

We obtained meteorological data from the North American Mesoscale (NAM) Analysis meteorological model^34^ because it includes all of the standard meteorological variables which play a role in PM_2.5_ levels, including planetary boundary layer height which can be important to help scale AOD values to ground-level estimates of PM_2.5_^35^. We calculated 24-hour averages from 6-hour average data for planetary boundary layer height, temperature at 2-meter elevation, relative humidity at 2 meters, dew point temperature at 2 meters, U- (east-west) and V- (north-south) components of wind speed at 10 meters, surface pressure, pressure reduced to mean sea level, vertical wind velocity at an altitude of 850 mb, and vertical wind velocity at an altitude of 700 mb. NAM has 12 km spatial resolution. The NAM data was retrieved using the rNOMADS package^36^.

PM_2.5_ concentration estimates from chemical transport models have been shown to be an important input to machine learning models for PM_2.5_^15,17^. We obtained daily estimates of PM_2.5_ at 12 km spatial resolution from runs of the CMAQ (Community Multi-scale Air Quality) model from the U.S. EPA for the years 2008–2016, the years for which the CMAQ estimates are available^37^.

The main reason that PM_2.5_ concentrations have been increasing in the western US, while they have been decreasing in other regions, is the increasing number and magnitude of wildfires in that region^7,8^. To include information on proximity to active fires in our machine learning models, we collected daily data about fire detection locations and size from the MODIS Thermal Anomalies/Fire Daily L3 Global 1 km product (MOD14A1 and MYD14A1)^38^. As fires in closer proximity are likely to influence PM_2.5_ more than fires farther away, we calculated the number of active fires in radial buffers of 25, 50, 100, and 500 km radii around each monitoring location, on the current day as well as on each of the previous seven days. We then calculated an inverse-distance-weighted average for each temporal lag. Finally, we created an indicator variable for whether there were one or more fires within 500 km of a monitor in the last week.

Elevation can influence PM_2.5_ concentrations. For example, PM_2.5_ can accumulate in mountain valleys during persistent cold air pools (commonly referred to as inversions) during winter^39^. We obtained elevation data from the 3D Elevation Program of the U.S. Geological Society, with has a resolution of 1 arc-second, which is approximately 30 m north/south and varies east/west with latitude^40^.

Surrounding land cover can be a proxy for air pollution emissions not from wildfires, such as emissions from traffic and industry in urban areas, and lack of particulate air pollution emissions in more vegetated areas. We used the land cover class information from the Landsat-derived National Land Cover Dataset (NLCD) 2011^41^, which has a spatial resolution of 30 m and uses circa 2011 Landsat satellite data to classify each pixel into one of a variety of land cover codes. We used the NLCD 2011 to calculate the percentage of urban development (codes 22, 23, and 24) within buffer radii of 1 km, 5 km, and 10 km around each monitor. We also included population density as areas with higher population have more sources of air pollution emissions. Population density was obtained from the 2010 U.S. Census^42^. For a time-varying measure of vegetation abundance, we used monthly measures of the Normalized Difference Vegetation Index (NDVI) from the MODIS satellite product MOD13A3^43^ which has a 1 km spatial resolution.

As another, potentially more specific proxy indicator of emissions from vehicles, we calculated the sum of all road lengths of type “Arterial” and “Collector” within 100, 250, 500, and 1000 m buffers of each monitoring location. Arterial roads are high-capacity urban roads, including highways. Collector roads are low-to-moderate capacity roads. The road data came from the National Highways Planning Network^44^, which contains spatial information on over 450,000 miles of highways in the United States.

To account for seasonality in PM_2.5_ data, we created the following predictor variables: cosine of day-of-week, cosine of day-of-year, and cosine of month. The use of cosine of these terms ensures that day/month values at the end and beginning of the week and year align.

We also created indicator variables to represent spatial and temporal variation in the data that could not be explained by any of the other spatial, temporal, or spatiotemporal variables. Use of nested levels of spatiotemporal variables can help to capture nonlinear spatiotemporal effects. Temporal variable nesting consisted of variables to indicate the periods 2008–2012, 2013–2016, and 2017–2018; year; season; cosine of month; and cosine of day of year. Spatial variable nesting consisted of dummy variables for region (within the 11 western states: northwest (i.e., WA, OR), southwest (i.e., CA, NV), four corners (i.e., AZ, CO, NM, UT), and northern mountain states (i.e., WY, MT, ID)); state; latitude; and longitude. We also included interaction terms for time period (grouping of years) and region. This type of nesting has been referred to as a “multiresolution basis”^45^.

### Data merging

We created seven datasets that all required merging the above datasets together: one dataset to train the model (with the 2008–2016 dataset able to be subset from the full 2008–2018 dataset) and six prediction datasets, with three spatial levels of prediction (county, ZIP code, and census tract) for each of the two models (2008–2016 which included CMAQ as a predictor variable, and 2008–2018 which did not include CMAQ as a predictor variable). The training dataset merged all predictor variables to each 24-hour average PM_2.5_ monitoring observation by linking the data temporally (using date) and spatially (by selecting the nearest spatial observation for each predictor variable using latitude and longitude). Similarly, the prediction datasets were created by spatially (by nearest latitude and longitude) and temporally (by date) linking all predictor variables to the centroid of each county, ZIP code, and census tract for each day in the study domain.

### Machine learning modelling

We employed ensemble machine learning to model PM_2.5_ exposures across the western US. Specifically, we used a generalized linear model (GLM) to combine the results from two machine learning algorithms: a random forest model and a gradient boosting model. These models performed best on preliminary analyses of random subsets of our dataset, which aligns with a previous study that found that tree-based models (using random forest, gradient boosting, and cubist algorithms) performed best in air pollution modelling^46^. Then, we used the same random subsets of the data to tune hyperparameters for each algorithm via a grid-search (Supplementary Information 1).

For the machine learning modelling, we performed both random and spatial 10-fold cross-validation so that our models would not overfit the training data^47^. We performed random 10-fold cross-validation, in which a random 10% of all observations are in each fold and the data is trained on 9 folds and tested on the left-out fold and then repeated 10 times such that every fold is a left-out fold once, for comparison to other studies. We performed spatial 10-fold cross-validation, whereby all observations from a given monitoring site are within the same fold, as a more appropriate tool for evaluating the accuracy of a model when predicting PM_2.5_ at new (left-out) locations, such as those in our prediction set^48^. Before either kind of 10-fold cross-validation, we also removed 10% of the monitoring locations for a testing data set, which was not used in model development. The spatial-folds analysis used a spatial held-out 10% (random 10% of the locations), while the random-folds analysis used a random held-out 10% (10% of the observations regardless of location). Most previous studies to create daily PM_2.5_ estimates using machine learning^15–18^ present results from only random 10-fold cross-validation, which violates the assumption of independence between folds because of repeated observations (on different days) from the same locations (PM_2.5_ monitor locations). Additionally, they do not provide performance metrics for predictions on a completely left-out test set. Their performance metrics are therefore overly optimistic for how their models will perform in their output data.

We used the metrics root-mean-squared error (RMSE) and R^2^ to report accuracy, for both the 10-fold cross-validation and for the left-out testing data set, for both spatial folds and random folds. Note that our spatial-folds metrics are different than spatial R^2^ that has been reported in other papers such as Di *et al*., (2019)^17^.

All analyses were run using R^49^, and all machine learning models utilized the R packages caret^50^ and caret ensemble^51^. Variable importance was calculated using the “permutation” importance algorithm in the caret package.

Our main model and prediction data set provide daily PM_2.5_ estimates by county, ZIP code, and census tract for 2008–2016. Because of our interest in the years 2017 and 2018, when there were many large wildfires in the western US, we created another model and prediction dataset for the 11-year period of 2008–2018 because we did not have CMAQ PM_2.5_ concentration output for 2017 and 2018. This separate ensemble machine learning model for 2008–2018 does not include CMAQ PM_2.5_ as a predictor variable but still created a consistent dataset of daily PM_2.5_ predictions for those 11 years.

We observed that our models, like most models, underpredict at very high values (e.g., above 200 µg/m^3^). We hypothesized that some of the higher values were being generated by a fundamentally different process than the lower values, most likely because of wildfires. We therefore did a sensitivity analysis in which we examined whether we would observe better performance by having different models for low values that are not likely influenced by wildfires and another for high values which are likely influenced by wildfires. This did not yield better performance and therefore these models did not create prediction datasets, but for the reader interested in this, a more detailed description of the split analysis (“high” versus “low”) can be found in Supplementary Information 2.

### Daily PM_2.5_ prediction creation

After merging the prediction input datasets by spatially and temporally linking all predictor variables to the centroid of each county, ZIP code, and census tract for each day in the study domain, we observed some missingness in the predictors that required imputation to create daily PM_2.5_ predictions. We observed missingness for fewer than 1% of the location-days within each state, except for the meteorological variables within Wyoming, for which nearly 10% of the location-days were missing. We used the missRanger^52^ package in R to impute the missing data for each state, based on all the available data for that state for all years in the given model (2008–2016 for the model including CMAQ PM_2.5_ and 2008–2018 otherwise).

Post-imputation, we applied the full models (trained with the entire training dataset including the left-out 10% testing data) to the prediction input datasets to make the final daily PM_2.5_ predictions at each county, ZIP code, and census tract within our 11-state study domain for the 9- and 11-year time periods.

We performed additional validation of our prediction data set that is separate from the accuracy estimates provided above of the machine learning model against the observations on which it was trained and tested. To assess the spatiotemporal alignment of our predicted values with observed values, we compared each observation to its spatially nearest prediction on the same day and calculated RMSE and R^2^ values for these. This is the most realistic estimate of our model’s performance overall.

## Data Records

Table 1 lists the names and descriptions of the datafiles that are available on figshare with a doi of 10.6084/m9.figshare.12568496^53^. We provide files by state for the predictions using the model with CMAQ PM_2.5_ (Ensemble_preds_with_CMAQ_[state].RData), and files by state for the predictions using the model without CMAQ PM_2.5_ (Ensemble_preds_no_CMAQ_[state].RData). All prediction data sets have predictions at three spatial resolutions: county, ZIP code, and census tract. Within the prediction data sets, the variable ranger_preds refers to the predictions made by the Ranger algorithm, the variable xgbt_preds to the predictions made by the extreme gradient boosting tree (XGBT) algorithm, and Ens_preds to the predictions made by the GLM ensemble of Ranger and XGBT. Missing_vars indicates if there was at least one missing input variable (and subsequent imputation) for that row of data.

**Table 1:** Data Files provided at 10.6084/m9.figshare.12568496^53^.

File Name(s)	Name of the Data Frame(s) in the .RData File	Years of Data	Variables
Ensemble_preds_no_CMAQ_[state].RData	DF	2008–2016	County_FIPS, Tract_code, ZCTA5_code, Lon, Lat, Date, ranger_preds, xgbt_preds, Missing_vars, Ens_pred
Ensemble_preds_with_CMAQ_[state].RData	DF	2008–2018	County_FIPS, Tract_code, ZCTA5_code, Lon, Lat, Date, CMAQ_ranger_preds, CMAQ_xgbt_preds, Missing_vars, Ens_pred

Because much of our input data derive from sources that are not ours, we cannot share out input data for our ML models at this time, but this data can be made available upon request. Most of our data sources are available free from government online databases, and therefore someone wishing to replicate our findings could download the data and follow our scripts, which we provide.

## Technical Validation

### Performance metrics on training and testing data

Table 2 shows the performance metrics (RMSE and R^2^) of our ensemble machine learning models with spatial folds and random folds. The results for the training data are based on 10-fold cross-validation, whereas the testing RMSE and R^2^ are for the completely left-out 10% of the data, a good test of how our model will do predicting at locations on which it did not train.

**Table 2:** Performance metrics (RMSE and R^2^) of our models using various subsets of data and types of CV folds.

Model	Training 10-fold CV RMSE (µg/m^3^) and R^2^	Testing (left out 10%) RMSE (µg/m^3^) and R^2^
2008–2016 with CMAQ, spatial folds	5.061 (0.659)	5.420 (0.589)
2008–2018 without CMAQ, spatial folds	6.576 (0.598)	6.599 (0.593)
2008–2016 with CMAQ, random folds	4.482 (0.732)	4.642 (0.715)
2008–2018 without CMAQ, random folds	5.482 (0.719)	5.954 (0.680)
2008 – 2016 without CMAQ, random folds*	4.747 (0.702)	
2008–2016 with CMAQ, no folds or testing set	1.726 (0.960)	
2008–2018 without CMAQ, full model, no folds or testing set	2.027 (0.961)	

*This test was performed to show the impact of including CMAQ in the model.

Overall, our models including CMAQ PM_2.5_ (2008–2016 models) perform better (have lower RMSE and higher R^2^ values) than models without CMAQ PM_2.5_ (2008–2018 models). This may be due to the additional information provided by the CMAQ output or could be because the models without CMAQ PM_2.5_ include two additional years of data that have more days with high PM_2.5_, which are much harder to predict accurately than lower values. For comparison, results for a random-folds model without CMAQ PM_2.5_ on the years 2008–2016 performed slightly worse than the model for years 2008–2016 with CMAQ PM_2.5_ included as a predictor variable.

The spatial-folds performance metrics are worse than the random-folds performance metrics. This is not surprising because the spatial folds do not allow for observations from the same location to be in multiple folds, therefore the models are predicting at locations that they did not train on, whereas random folds have likely trained on observations at all locations, thus are more likely to predict values better for those locations. Using solely random folds can therefore be misleading as to the performance of the models given that these kinds of models predict at locations without monitoring data and thus not in the training data^48^. Thus, we posit that most of the models presented in the literature previously that use random folds CV are reporting R^2^ values that are likely higher than their predictive performance at non-sampled locations. Henceforth all results in this section refer to the spatial-folds analysis.

Performance of our models on our completely left-out testing data set provide worse metrics than their training (10-fold CV) counterparts. Some of the discrepancy between training and testing set results is because the testing data set was not used to inform the development of the model; some of the discrepancy is because of random chance of a given monitoring site being in the testing data set. Given that most previous machine learning air pollution modelling studies do not report metrics for completely left-out testing data^15–19^, their results on the accuracy of their models’ predictions may be misleading. For comparison, the performance metrics for our full models (without any cross-validation folds) on the 2008–2016 and the 2008–2018 datasets are, respectively, RMSE = 1.726 µg/m^3^ and R^2^ = 0.960; RMSE = 2.027 µg/m^3^ and R^2^ = 0.961. These are much better performance metrics than any of those in Table 2 because all models overfit the data they are trained on. Full models train on all observations with none left out and therefore are not realistic representations of how accurately the model will predict at locations outside of the training set.

**Fig. 3:**
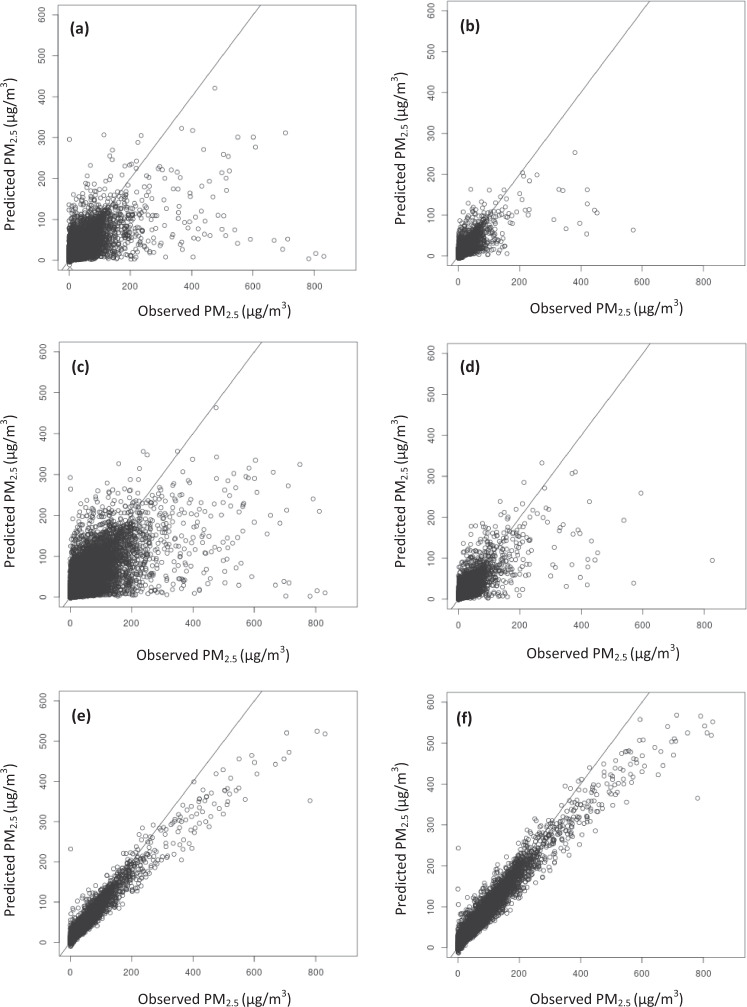
Predicted-versus-observed plots of daily PM_2.5_ values from our ensemble machine learning models using spatial 10-fold cross validation. All lines in Fig. 3 depict the y = x lines in which predictions are equal to observed values. The subplots refer to (**a**) spatial 10-fold cross-validation training dataset for the 2008–2016 model (90% of the data) (includes CMAQ PM_2.5_), (**b**) spatial 10-fold cross-validation testing dataset (10% of the data) for the 2008–2016 model (includes CMAQ PM_2.5_), (**c**) spatial 10-fold cross-validation training dataset for the 2008–2018 model (90% of the data) (does not include CMAQ PM_2.5_), (**d**) spatial 10-fold cross-validation testing dataset (10% of the data) for the 2008–2018 model (does not include CMAQ PM_2.5_), (**e**) all observations in the 2008–2016 model (includes CMAQ PM_2.5_) with no CV (the full model), (**f**) all observations in the 2008–2018 model (does not include CMAQ PM_2.5_) with no CV (the full model).

The predicted-versus-observed plots in Fig. 3 demonstrate that the model trained on all of the data (Fig. 3e,f) has the greatest agreement between predictions and observations, which is expected when machine learning models are fit without cross-validation because they overfit to the data used. These figures also show that there were many more high values in the years 2017 and 2018 (on the non-CMAQ model plots, Fig. 3c,d,f). Also, all models tend to underpredict values of PM_2.5_ higher than 200 µg/m^3^, which is likely because there are fewer high values than low values in the training set. Many previous studies using similar methodology only present the accuracy of their PM_2.5_ predictions up to 60 µg/m^3^ without documenting the range of values trained on^16,17^ or training on observations that had a much smaller range (up to 136.8 µg/m^3^)^18^. All of these models additionally perform worse at high concentrations, similar to ours, due to few observations to train on at those values. The Di *et al*., (2016)^16^ and Di *et al*., (2019)^17^ papers dismiss the importance of their models’ poor performance at values above 60 µg/m^3^ because they state that there are few days in locations in the U.S. that experience PM_2.5_ concentrations above that value. This implies that days affected by wildfires are either not important to predict well and/or that these previous models should not be used to predict PM_2.5_ on days in locations affected by wildfire smoke. All of these previous models were trained solely on EPA AQS without inclusion of the additional monitoring data from the USFS and other sources that we included so that our models would have those higher values on which to train. Although we do show underprediction at these higher values, training our model on a larger range of input values than previous studies allows us to predict values that are likely closer to the “true” concentration to which populations were exposed. It should be noted the two Di *et al*. papers^16,17^ only present performance metrics for all values below 60 µg/m^3^ and do not indicate what the range of values were that were used to train their model. The Hu *et al*. (2017)^18^ and Park *et al*. (2019)^19^ models were only trained on values up to 136.8 µg/m^3^.

Table 3 shows the RMSE (and R^2^) values of our models on different levels of PM_2.5_, years, states, and seasons using *spatial folds*. RMSE (and R^2^) values from the *random-folds analysis* of the same models are in Supplementary Table 2.

**Table 3:** RMSE and R^2^ values for the 10-fold spatial cross-validation training set (90% of the data) and the 10% left-out testing set for both the 2008–2016 and 2008–2018 ensemble models.

	2008–2016 Ensemble Model Training RMSE (µg/m^3^) (and R^2^)	2008–2016 Ensemble Model Testing RMSE (µg/m^3^) (and R^2^)	2008–2018 Ensemble Model Training RMSE (µg/m^3^) (and R^2^)	2008–2018 Ensemble Model Testing RMSE (µg/m^3^) (and R^2^)
**By PM**_**2**.**5**_ **Level (µg/m**^**3**^**)**				
Below 35	3.346 (0.682)	3.905 (0.575)	4.201 (0.523)	4.151 (0.522)
Below 60	3.664 (0.714)	4.179 (0.612)	4.651 (0.568)	4.499 (0.569)
Below 150	4.010 (0.720)	4.526 (0.621)	5.154 (0.615)	5.009 (0.612)
Below 300	4.319 (0.706)	4.646 (0.631)	5.635 (0.626)	5.376 (0.641)
Below 500	4.618 (0.690)	5.247 (0.603)	6.044 (0.618)	6.118 (0.616)
Below 1000	5.061 (0.659)	5.420 (0.589)	6.576 (0.598)	6.599 (0.593)
**By Year**				
2008	4.037 (0.787)	4.818 (0.689)	4.920 (0.680)	4.903 (0.661)
2009	3.755 (0.744)	4.416 (0.637)	4.603 (0.614)	4.611 (0.602)
2010	3.537 (0.707)	3.834 (0.634)	4.372 (0.559)	4.013 (0.579)
2011	4.016 (0.723)	4.264 (0.642)	4.840 (0.598)	4.396 (0.615)
2012	5.459 (0.684)	8.006 (0.504)	6.180 (0.581)	8.456 (0.449)
2013	4.990 (0.683)	5.927 (0.588)	5.836 (0.562)	6.022 (0.576)
2014	4.816 (0.668)	5.175 (0.572)	5.728 (0.530)	5.359 (0.540)
2015	5.881 (0.667)	5.107 (0.687)	6.723 (0.571)	5.423 (0.645)
2016	7.239 (0.449)	5.296 (0.520)	7.794 (0.370)	5.627 (0.454)
2017	N/A	N/A	9.348 (0.684)	8.868 (0.669)
2018	N/A	N/A	8.553 (0.687)	10.435 (0.619)
**By State**				
Arizona	3.139 (0.614)	4.094 (0.374)	3.795 (0.469)	3.894 (0.418)
California	4.753 (0.719)	4.026 (0.756)	6.577 (0.632)	5.197 (0.724)
Colorado	5.940 (0.499)	3.539 (0.472)	9.143 (0.351)	3.758 (0.432)
Idaho	7.016 (0.663)	11.539 (0.546)	7.789 (0.605)	10.034 (0.607)
Montana	5.428 (0.648)	5.192 (0.593)	7.642 (0.636)	7.183 (0.595)
Nevada	3.639 (0.679)	3.630 (0.634)	4.273 (0.497)	3.809 (0.568)
New Mexico	3.628 (0.536)	9.732 (0.028)	3.732 (0.388)	10.823 (0.006)
Oregon	5.239 (0.597)	10.063 (0.362)	8.441 (0.612)	12.081 (0.436)
Utah	4.667 (0.638)	4.979 (0.647)	5.223 (0.495)	4.584 (0.665)
Washington	5.642 (0.583)	4.409 (0.586)	6.814 (0.529)	8.412 (0.515)
Wyoming	6.983 (0.516)	3.859 (0.531)	6.405 (0.418)	3.839 (0.465)
**By Season**				
Fall	5.960 (0.598)	6.465 (0.520)	7.896 (0.599)	8.505 (0.561)
Spring	3.168 (0.611)	3.227 (0.561)	3.871 (0.444)	3.293 (0.543)
Summer	5.745 (0.635)	5.760 (0.571)	7.771 (0.605)	7.629 (0.591)
Winter	4.682 (0.767)	5.632 (0.658)	5.787 (0.639)	5.620 (0.650)

The models with CMAQ PM_2.5_ (2008–2016 models) always perform better than the models without CMAQ PM_2.5_ (2008–2018 models). This is likely because of the importance of PM_2.5_ concentration data from a chemical transport model in the machine learning models (Supplementary Table 5). Although chemical transport models provide spatiotemporal estimates of PM_2.5_, they do not always predict accurately and many have shown that empirical models like the ones presented here can improve predictive performance of chemical transport models. However, the algorithms we used perform well even when one important predictor is missing. When CMAQ PM_2.5_ is not included, MAIAC AOD rises in variable importance (Supplementary Table 5). Although collinearity between variables does not matter for prediction with random forest, it most likely reduces the variable importance calculations via permutation^54^.

**Fig. 4:**
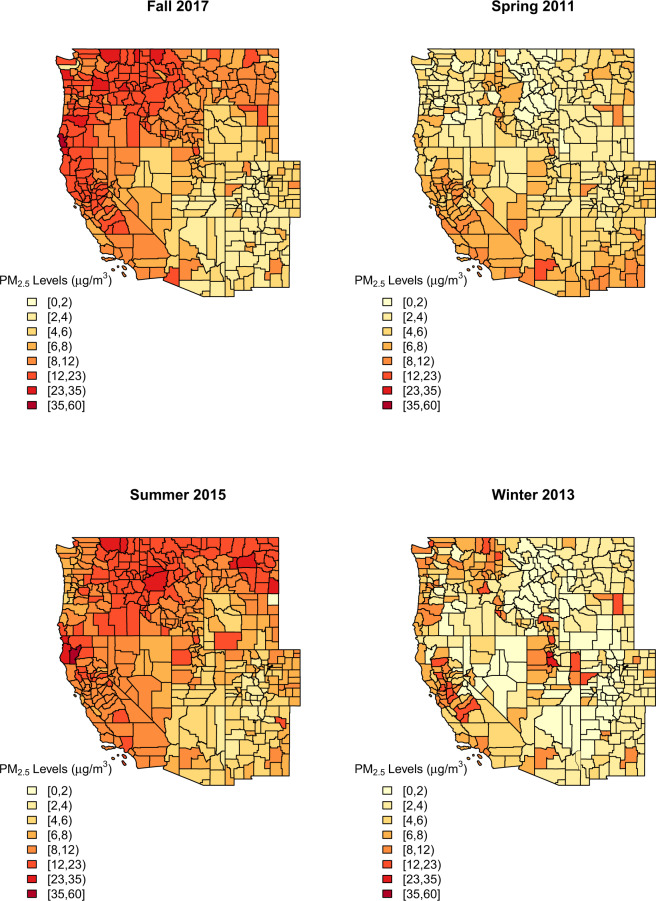
Map of seasonal average predicted PM_2.5_ by county for selected seasons using the 2008–2018 model. Winter = December, January, February; Spring = March, April, May; Summer = June, July, August; Fall = September, October, November.

We have better predictive performance at lower levels of PM_2.5_. This is likely partially because a much larger number of observations at lower values allowed the model to be better trained at those values; it is well-known that models tend to have greater trouble predicting extremes. In the spatiotemporal subsets, we observed higher RMSE for the years 2012 and 2015–2018, which have some of the highest PM_2.5_ values due to high numbers of wildfires in our study domain during those years. The patterning of results by state is less clear, although it is notable that the RMSE values for California are lower than might be expected given the state’s higher-than-average PM_2.5_ levels. This is likely because over 40% of our monitoring locations and thus observations are from California. Finally, the RMSE values for Spring are lower than those from the other seasons, which is likely due to the predominance of lower PM_2.5_ values in the spring (Fig. 4). One thing to note related to the performance metrics in Table 3 is that sometimes the R^2^ values are quite low for the testing data for a given year or state because there were few observations within that state or year within the pre-selected 10% testing hold-out done across all of the data before the modelling; this is not a 10% of observations from each state or year.

We also provide RMSE values normalized by the mean of each corresponding dataset in Supplementary Tables 3 and 4 (corresponding to the spatial and random folds results respectively). These tables provide a sense of the predictive performance relative to the observed values. We observe that the model has larger error relative to the mean of the observed data for years and seasons with more wildfires. This is unsurprising given that squaring the errors in the RMSE calculation tends to weight large differences more heavily than the simple sum in the mean calculation. There is less of a clear pattern for the normalized RMSE by state, though the fact that our model does relatively well in California is likely due to having more air quality monitors and thus training data there. The spatial folds and random folds results follow the same overall pattern, though the random folds results have smaller errors compared to their mean values, likely because there is training data from all locations in all folds yet use of these metrics would lead to overconfident predictions in locations without monitoring data.

### Prediction data set validation

Descriptive statistics for the predicted PM_2.5_ values by geographic scale (county, ZIP code, and census tract) are provided in Supplementary Table 6 (for the 2008–2016 model) and Supplementary Table 7 (for the 2008–2018 model). Our metrics for comparing our model predictions to nearby observed monitors are shown in Table 4. When we consider only stable monitors (excluding monitors with less than 31 days of observations, 1% of the training data), the 2008–2018 model yields an RMSE of 5.6 μg/m^3^ and an R^2^ of 0.581; the 2008–2016 model yields an RMSE of 4.2 μg/m^3^ and an R^2^ of 0.696. When we include all observations, the 2008–2018 model yields an RMSE of 6.2 μg/m^3^ and an R^2^ of 0.643; the 2008–2016 model yields an RMSE of 5.1 μg/m^3^ and an R^2^ of 0.647.

**Table 4:** Performance metrics (RMSE and R^2^) comparing model predictions with the closest nearby PM_2.5_ monitoring observations.

Model	Compared to all monitors RMSE (μg/m^3^) (and R^2^)	Compared to stable monitors (excluding monitors with less than 31 days of observations, 1% of the training data) RMSE (μg/m^3^) (and R^2^)
2008–2016 model	5.1 (0.647)	4.2 (0.696)
2008–2018 model	6.2 (0.643)	5.6 (0.581)

**Fig. 5:**
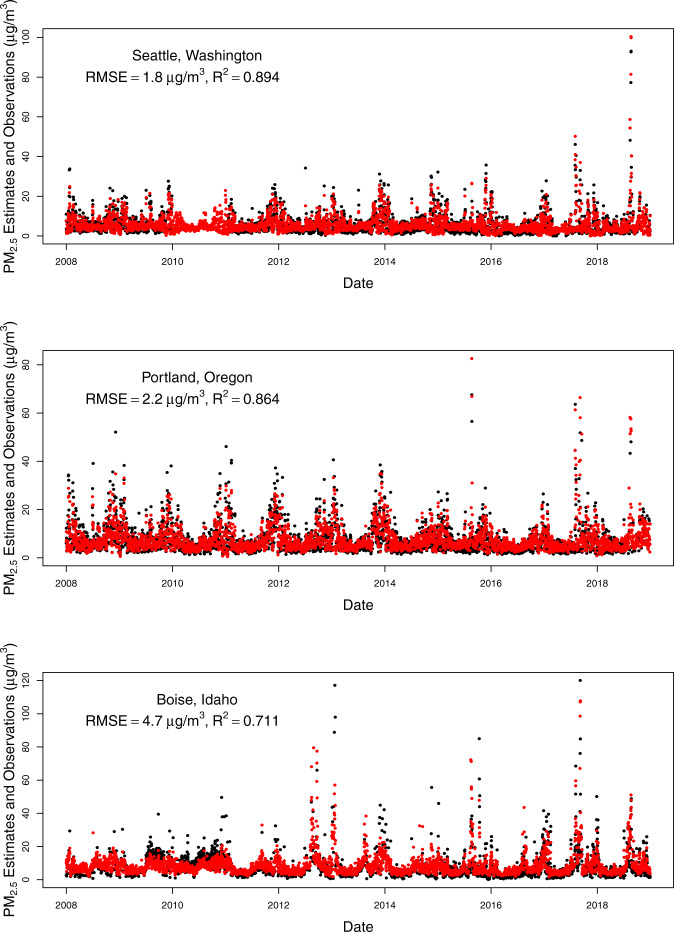
Time series of daily PM_2.5_ estimates (red) at the nearest prediction locations (centroid of census tract, ZIP code, or county) near daily PM_2.5_ monitoring observations (black) for Seattle, WA, Portland, OR, and Boise, ID using the 2008–2018 models (with RMSE and R^2^ values for that location).

**Fig. 6:**
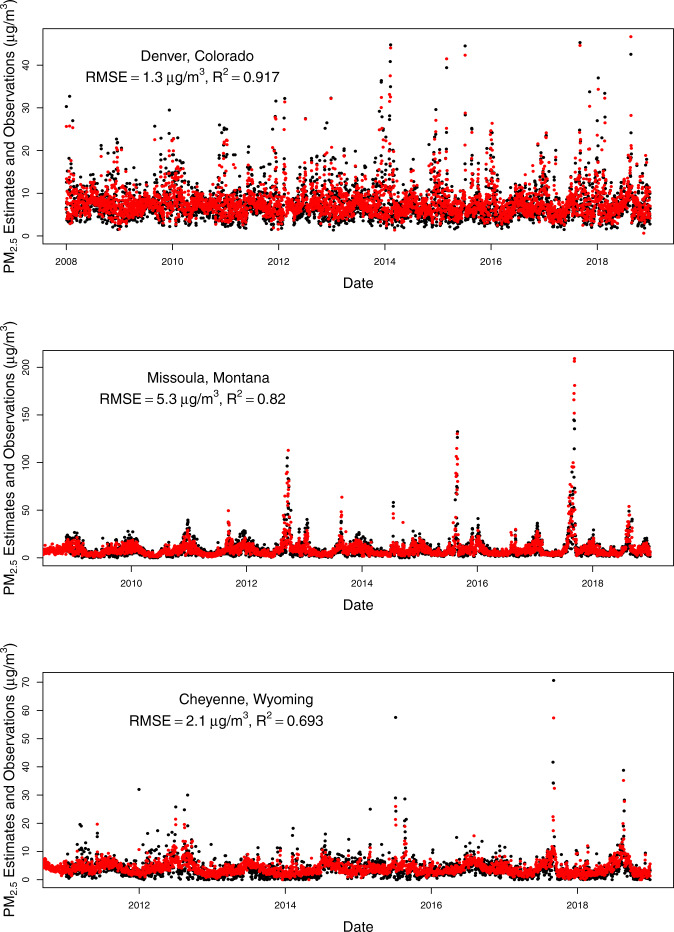
Time series of daily PM_2.5_ estimates (red) at the nearest prediction locations (centroid of census tract, ZIP code, or county) near daily PM_2.5_ monitoring observations (black) for Denver, CO, Missoula, MT, and Cheyenne, WY using the 2008–2018 models (with RMSE and R^2^ values for that location).

**Fig. 7:**
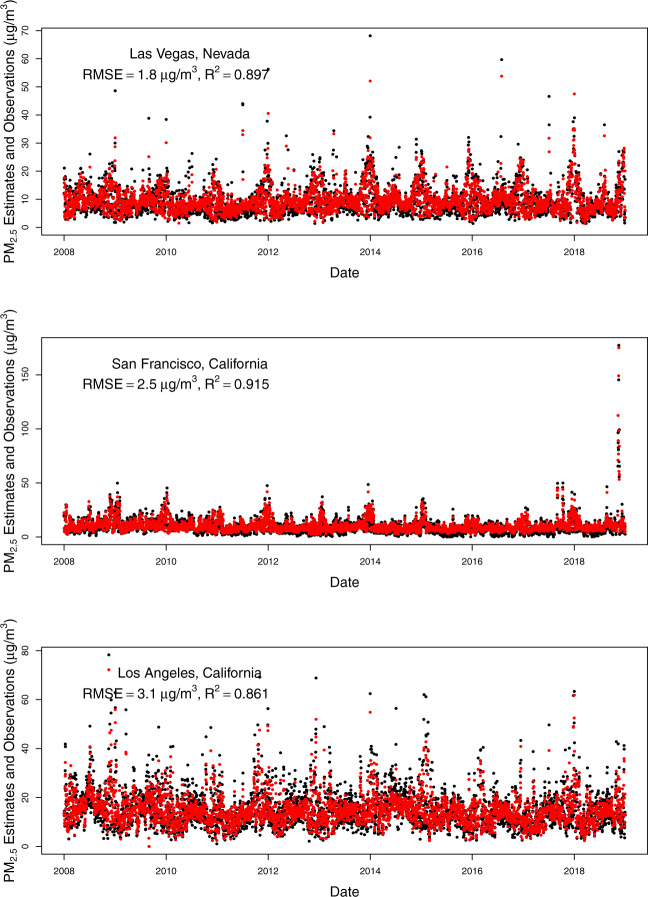
Time series of daily PM_2.5_ estimates (red) at the nearest prediction locations (centroid of census tract, ZIP code, or county) near daily PM_2.5_ monitoring observations (black) for Las Vegas, NV, San Francisco, CA, and Los Angeles, CA using the 2008–2018 models (with RMSE and R^2^ values for that location).

**Fig. 8:**
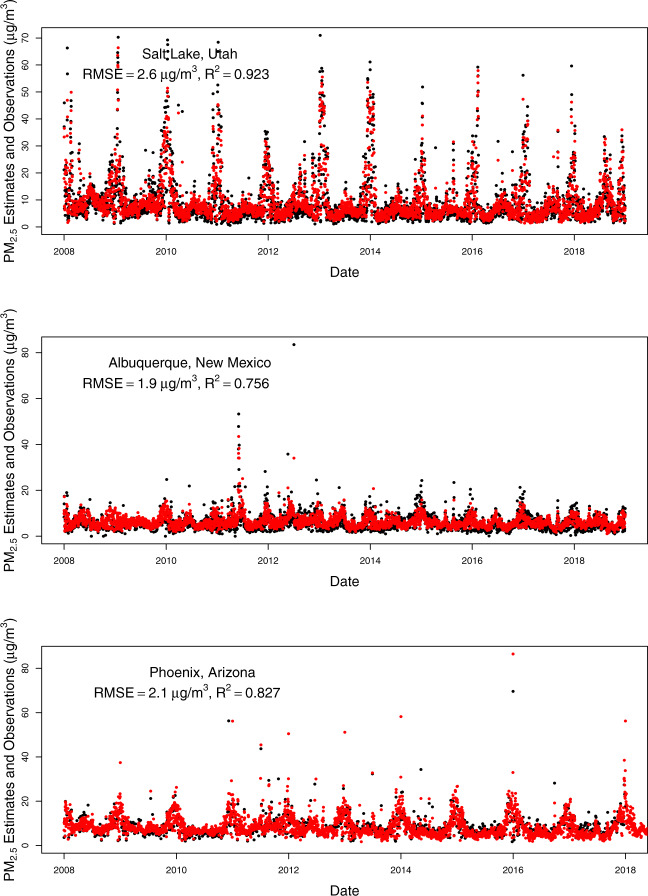
Time series of daily PM_2.5_ estimates (red) at the nearest prediction locations (centroid of census tract, ZIP code, or county) near daily PM_2.5_ monitoring observations (black) for Salt Lake City, UT, Albuquerque, NM, and Phoenix, AZ using the 2008–2018 models (with RMSE and R^2^ values for that location).

To assess the temporal alignment of our predicted values with observed values, we plotted time series for predicted daily PM_2.5_ for a monitoring station within a major city in each state of our study domain compared to observations from the closest monitors (Figs. 5–8). The monitoring stations were selected for having the most continuous monitoring observations in a large city within each state, but we included two large cities for California because of its large population and geographic variability with regards to air pollution. From these plots, you can see that our model predictions depict the temporal variability in PM_2.5_ concentrations at monitoring stations nicely including the high values when they occur (most often during wildfire seasons) even for cities in which the air pollution during the most recent wildfire seasons have dwarfed the PM_2.5_ levels in previous wildfire seasons (e.g., San Francisco, Seattle). There is quite good agreement between the predictions (which are from the nearest prediction location (centroid of census tract, ZIP code, or county) to each selected monitoring location (R^2^ values range from 0.693 in Cheyenne, WY to 0.923 in Salt Lake City, UT, with all but three above 0.80). The RMSE values also vary, but these are more impacted by the range of PM_2.5_ values observed at a given location. The models predict worse at higher values, thus locations with more PM_2.5_ variability (mostly driven by high PM_2.5_ values during wildfire seasons) tend to have higher RMSE values (e.g., Missoula, Montana). It is important to note that these are predictions in our prediction data set (either census tract, ZIP code, or county centroid) that are nearest to the monitor and not at that exact location. In particular, the distances from the nearest prediction to the monitor in Santa Fe, New Mexico and Boise, Idaho were nearly an order of magnitude larger than the distances for each of the other cities, which contributed to their lower R^2^ and higher RMSE values.

We also mapped seasonal averages of our daily PM_2.5_ predictions by county to assess the spatial patterning in the predictions and whether they aligned with the spatial pattern in the monitored data (Fig. 4). The year picked for each season was to demonstrate our model’s ability to predict the spatial pattern of PM_2.5_ during specific times when PM_2.5_ was particularly high in certain regions such as the large wildfires in Arizona in the spring of 2011, in the North in summer 2015, in the Pacific Northwest and California in the Fall of 2017, and to show the PM_2.5_ from inversions in Utah and California in the winter of 2013. Not all counties had monitoring data, so it was not possible to plot the monitoring data in the same way. Overall, these maps show lower PM_2.5_ levels in the spring; Spring 2011 is just one example of this. In the winter, in the western US counties, many areas experience quite low levels of PM_2.5_, but there are some regions that have higher levels due to wintertime inversions (e.g., Salt Lake City, California’s Central Valley). We also chose to highlight two seasons with high wildfire activity: the summer of 2015 when there were many wildfires burning in the Pacific northwest and the fall of 2017 when California had a large number of wildfires. We note that, as these are seasonal averages, the PM_2.5_ levels are not as high as the maximum daily predicted levels for each county.

## Usage Notes

All of our data files are available in the RData format. An RData file can be opened in R using the “load” command. Note that the state data frames range in size from about 2 million rows to about 34 million rows. A computer with lots of memory and/or cloud computing may be necessary to analyse these data.

Because the predictive model is continuous and not confined to positive numbers, low predictions (close to 0) will sometimes go negative. Because this is physically unrealistic, we recommend replacing the few negative PM_2.5_ predictions with 0, as we have done to generate the plots and performance metrics shown in the Technical Validation section.

Then, our PM_2.5_ estimates may be merged with health data or other data at the county, ZIP code, and census tract levels based on FIPS codes (for county and census tract) and ZCTA5 codes (for ZIP code). If a research team is concerned about our imputation technique, then they may wish to exclude any rows that have one or more missing variables.

## Supplementary information

Supplementary Material

## Data Availability

All code used for downloading and processing the data used in this project, including the machine learning and technical validation code, may be accessed at 10.5281/zenodo.4499264^55^. To ensure that our work is reproducible, all code is written in open-source languages. Some scripts are in R and others are in Python. Python scripts need Python 3; R versions beyond 3.5.1 should suffice. The scripts on Zenodo are a copy of our GitHub repository of scripts and contains the following files and directories: • **General_Project_Functions**: Scripts to obtain the prediction set locations and tools that are generally useful during the data processing, such as making buffers around points and reprojecting point coordinates. • **Get_PM25_Observations**: Scripts to process PM_2.5_ observations from across the western U.S. These observations are used to train our machine learning models. • **Get_Earth_Observations**: Scripts to download and process observations from data sets that are used both as inputs for our machine learning models during training and as inputs for our models in the prediction stage. The file *Overall_steps* provides all necessary directions. Individual *README* files (in each folder) provide more details if there are any. • **Merge_Data**: Scripts to merge all the data together and derive some spatio-temporal variables. • **Machine_Learning**: Scripts to run and evaluate our machine learning models. The folder Final_scripts contains all code used for our final analysis. The code in the Exploring_models folder was all preliminary testing. • **Estimate_PM25**: Scripts to use our machine learning models to make final predictions and to explore the prediction data sets over time and space.

## Online-only Table

**Online-only Table 1:** Input variables and their descriptions/sources and names in the training/validation and prediction datasets.

Variable	Source/Description
**Spatial variables**	
Coordinates in degrees (Longitude, Latitude)	
State	
Region within the western US	Northwest = WA, OR; Southwest = CA, NV; Four corners = AZ, CO, NM, UT; and Northern mountain states = WY, MT, ID
Population Density	US Census 2010^44^; spatial resolution = census tract level
Percent urban landcover in circular buffers of radius 1 km, 5 km, 10 km (%)	Derived from the USGS National Map Program NLCD, derived from 2011 Landsat imagery^43^
Lengths of arterial and collector roads in circular buffers of radius 100 m, 250 m, 500 m, 1000 m	Derived from the US Federal Highway Administration National Highway Planning Network^46^
Elevation (m)	USGS 3D Elevation Program, National Elevation Database (NED)^42^
**Temporal variables**	
Date	
Season	Summer = June-August, Fall = September-November, Winter = December-February, Spring = March-May
Cosine of month	Cosine (2*pi*month/12)
Cosine of day of year	Cosine (2*pi*day of year/365)
Cosine of day of week	Cosine (2*pi*day of week/7)
Day of week	
Year	
Study period – binary indicator of middle study period	Years 2013–2016
Study period – binary indicator of late study period	Years 2017–2018
**Spatio-temporal variables**	
PM_2.5_ observations (µg/m^3^)	Federal and state air quality databases
Aerosol optical depth	NASA MAIAC^29^; spatial resolution = 1 km, temporal resolution = generally two observations per day are found but additional ones are possible near the edges of the scan
Chemical transport model simulation	US EPA CMAQ^38^; spatial resolution = 12 km, temporal resolution = daily, only available for 2008–2016
Vegetation index	NASA MODIS NDVI^45^; spatial resolution = 1 km, temporal resolution = monthly
Active fire points within the last week, weighted inversely by distance (up to 500 km)	Derived from NASA MODIS thermal anomalies^56^; spatial resolution = 1 km, temporal resolution = daily
Indicator for whether there was at least one fire in the last week within 500 km	Derived from NASA MODIS thermal anomalies^56^; spatial resolution = 1 km, temporal resolution = daily
Temperature 2 m above the ground (K)	North American Mesoscale Analysis (NAM)^35^; spatial resolution = 12 km, temporal resolution = 6 hours
Relative humidity 2 m above the ground (%)	North American Mesoscale Analysis (NAM)^35^; spatial resolution = 12 km, temporal resolution = 6 hours
Dew point temperature 2 m above the ground (K)	North American Mesoscale Analysis (NAM)^35^; spatial resolution = 12 km, temporal resolution = 6 hours
Height of the planetary boundary layer (m)	North American Mesoscale Analysis (NAM)^35^; spatial resolution = 12 km, temporal resolution = 6 hours
Surface pressure (Pa)	North American Mesoscale Analysis (NAM)^35^; spatial resolution = 12 km, temporal resolution = 6 hours
Pressure reduced to mean sea level (Pa)	North American Mesoscale Analysis (NAM)^35^; spatial resolution = 12 km, temporal resolution = 6 hours
East-West and North-South components of windspeed 10 m above the ground (m/s)	North American Mesoscale Analysis (NAM)^35^; spatial resolution = 12 km, temporal resolution = 6 hours
Vertical component of windspeed, 700 and 850 mb (m/s)	North American Mesoscale Analysis (NAM)^35^; spatial resolution = 12 km, temporal resolution = 6 hours
Interaction indicators between region and time span	4 regions (see above), 3 timespans(see above)
