# Supplementary material for: Daily PM2.5 concentration estimates by county, ZIP code, and census tract in 11 western states 2008–2018
Source: Sci Data. 2021 Apr 19;8:112. doi: 10.1038/s41597-021-00891-1 (PMC8055869; doi:10.1038/s41597-021-00891-1)
Supplement: Supplementary file 1 — Supplementary Material [file 41597_2021_891_MOESM1_ESM.docx]

**Supplementary Material for “Daily PM2.5 concentration estimates by county, ZIP code, and census tract in 11 western states 2008-2018** *” by Reid, Considine, Maestas, and Li.*

Contents

[Supplementary Information 1: Final hyperparameters used in the machine learning models 2](#_Toc63324410)

[Supplementary Information 2: Description and results of the high versus low models 2](#_Toc63324411)

[Supplementary Table 1. Descriptive statistics for PM_2.5_ monitoring observations for 2008-2018 3](#_Toc63324412)

[Supplementary Table 2. RMSE and R^2^ values for the 10-fold random folds cross-validation training set and the 10% left-out testing set for both the 2008-2016 and 2008-2018 ensemble models 5](#_Toc63324413)

[Supplementary Table 3. RMSE values for the spatial folds results (shown in Table 3 in the main text) normalized (divided) by the mean of each corresponding observation subset. 7](#_Toc63324414)

[Supplementary Table 4. RMSE values for the random folds results (shown in Table 3 in the main text) normalized (divided) by the mean of each corresponding observation subset. 9](#_Toc63324415)

[Supplementary Table 5: Variable Importance metrics by model 11](#_Toc63324416)

[Supplementary Table 6: Descriptive statistics for predicted daily PM_2.5_ from the 2008-2016 ensemble model 13](#_Toc63324417)

[Supplementary Table 7: Descriptive statistics for predicted daily PM_2.5_ from the 2008-2018 ensemble model 15](#_Toc63324418)

# Supplementary Information 1: Final hyperparameters used in the machine learning models

For Ranger: .mtry = 15, .splitrule = "variance", .min.node.size = 6

For XGBTree: .nrounds = 150, .max_depth = 3, .eta = 0.3, .gamma = 0, .colsample_bytree = 2/3, .min_child_weight = 1, .subsample = 1

# Supplementary Information 2: Description and results of the high versus low models

We developed a preliminary classification model to split the data into “high” versus “low” values, with 15µg/m^3^ being the most plausible and accurate cut-point. Here, plausibility refers to the fact that with a split of 15 µg/m^3^or higher, variables associated with wildfires began ranking in the top 20 most important variables (calculated with the “permutation” importance algorithm). After developing this preliminary classification model, we pursued two approaches to develop regression models for the data classified as “high” or “low”. The first was to train the “high” and “low” regression models on data classified as “high” and “low”, respectively. The second was to train the “high” and “low” regression models on data that we knew to be greater than or less than 15 µg/m^3^ respectively. Ultimately, we found that these splitting approaches did not improve our predictions at higher levels of PM_2.5_. In fact, they only slightly improved predictions at lower levels of PM_2.5._ Thus, we decided to use the overall models (not splitting into “high” or “low”) for the final analysis.

# Supplementary Table 1. Descriptive statistics for PM_2.5_ monitoring observations for 2008-2018

| **Subset** | **N** | **Mean (µg/m^3^)** | **Min (µg/m^3^)** | **Q1 (µg/m^3^)** | **Median (µg/m^3^)** | **Q3 (µg/m^3^)** | **Max (µg/m^3^)** |
| --- | --- | --- | --- | --- | --- | --- | --- |
| **Year** |  |  |  |  |  |  |  |
| 2008 | 121,396 | 8.998 | 0 | 3.8 | 6.6 | 11.4 | 200.200 |
| 2009 | 131,397 | 8.329 | 0 | 3.7 | 6.3 | 10.6 | 195.583 |
| 2010 | 143,040 | 7.441 | 0 | 3.3 | 5.7 | 9.6 | 114.000 |
| 2011 | 147,828 | 8.316 | 0 | 3.5 | 6.2 | 10.6 | 208.025 |
| 2012 | 156,946 | 8.156 | 0 | 3.6 | 6.2 | 10.2 | 705.458 |
| 2013 | 163,096 | 8.559 | 0 | 3.7 | 6.5 | 10.6 | 452.792 |
| 2014 | 162,365 | 7.850 | 0 | 3.5 | 6.0 | 9.8 | 504.542 |
| 2015 | 137,573 | 7.648 | 0 | 3.2 | 5.5 | 9.2 | 830.792 |
| 2016 | 140,581 | 6.929 | 0 | 3.0 | 5.2 | 8.7 | 804.500 |
| 2017 | 150,213 | 8.938 | 0 | 3.2 | 5.7 | 10.0 | 811.792 |
| 2018 | 137,098 | 9.193 | 0 | 3.6 | 6.0 | 10.0 | 826.292 |
| **State** |  |  |  |  |  |  |  |
| Arizona | 79,964 | 6.365 | 0 | 3.3 | 5.3 | 7.9 | 199.300 |
| California | 680,549 | 10.269 | 0 | 5.0 | 8.2 | 12.6 | 791.625 |
| Colorado | 61,453 | 5.66 | 0 | 2.3 | 4.5 | 7.3 | 781.455 |
| Idaho | 59,456 | 7.949 | 0 | 3.0 | 5.3 | 8.9 | 519.391 |
| Montana | 90,837 | 7.431 | 0 | 2.5 | 4.8 | 8.3 | 641.900 |
| Nevada | 39,311 | 6.829 | 0 | 3.5 | 5.7 | 8.4 | 230.000 |
| New Mexico | 63,717 | 5.231 | 0 | 2.5 | 4.2 | 6.6 | 263.000 |
| Oregon | 150,682 | 7.5 | 0 | 3.1 | 4.8 | 8.2 | 811.792 |
| Utah | 83,290 | 7.202 | 0 | 3.3 | 5.2 | 8.2 | 225.250 |
| Washington | 230,120 | 6.546 | 0 | 3.0 | 4.7 | 7.6 | 830.792 |
| Wyoming | 51,128 | 4.311 | 0 | 1.8 | 3.2 | 5.2 | 513.417 |
| **Season** |  |  |  |  |  |  |  |
| Fall | 399,121 | 8.643 | 0 | 3.7 | 6.4 | 10.5 | 830.792 |
| Spring | 399,129 | 6.144 | 0 | 3.1 | 5.0 | 7.9 | 189.900 |
| Summer | 405,596 | 8.76 | 0 | 4.0 | 6.4 | 10.3 | 712.917 |
| Winter | 387,687 | 9.299 | 0 | 3.0 | 6.5 | 12.2 | 557.000 |

# Supplementary Table 2. RMSE and R^2^ values for the 10-fold random folds cross-validation training set and the 10% left-out testing set for both the 2008-2016 and 2008-2018 ensemble models

|  | **2008-2016 Ensemble Model Training RMSE (µg/m3) (and R2)** | **2008-2016 Ensemble Model Testing RMSE (µg/m3) (and R2)** | **2008-2018 Ensemble Model Training RMSE (µg/m3) (and R2)** | **2008-2018 Ensemble Model Testing RMSE (µg/m3) (and R2)** |
| --- | --- | --- | --- | --- |
| **By PM_2.5_ Level (µg/m3)** | | | | |
| Below 35 | 2.792 (0.779) | 2.775 (0.783) | 3.165 (0.729) | 3.213 (0.726) |
| Below 60 | 3.071 (0.8) | 3.013 (0.807) | 3.548 (0.753) | 3.572 (0.752) |
| Below 150 | 3.407 (0.798) | 3.305 (0.81) | 4.043 (0.767) | 4.021 (0.768) |
| Below 300 | 3.725 (0.781) | 3.451 (0.802) | 4.509 (0.762) | 4.337 (0.77) |
| Below 500 | 4.058 (0.762) | 3.859 (0.781) | 4.945 (0.745) | 4.981 (0.738) |
| Below 1000 | 4.482 (0.732) | 4.642 (0.715) | 5.482 (0.719) | 5.954 (0.68) |
| **By Year** | | | | |
| 2008 | 3.369 (0.853) | 3.454 (0.852) | 3.891 (0.809) | 3.333 (0.86) |
| 2009 | 3.206 (0.816) | 2.771 (0.856) | 3.678 (0.767) | 2.85 (0.848) |
| 2010 | 2.901 (0.8) | 2.619 (0.835) | 3.195 (0.764) | 2.639 (0.832) |
| 2011 | 3.377 (0.802) | 2.985 (0.854) | 3.665 (0.772) | 3.029 (0.851) |
| 2012 | 4.995 (0.746) | 5.395 (0.675) | 5.129 (0.732) | 6.132 (0.581) |
| 2013 | 4.398 (0.754) | 3.856 (0.807) | 4.606 (0.733) | 3.85 (0.807) |
| 2014 | 4.229 (0.741) | 3.974 (0.761) | 4.693 (0.686) | 4.529 (0.693) |
| 2015 | 5.275 (0.733) | 4.441 (0.772) | 5.643 (0.697) | 4.925 (0.719) |
| 2016 | 6.493 (0.52) | 8.859 (0.397) | 6.623 (0.499) | 8.559 (0.449) |
| 2017 | N/A | N/A | 8.243 (0.75) | 9.239 (0.683) |
| 2018 | N/A | N/A | 7.382 (0.768) | 9.925 (0.639) |
| **By State** | | | | |
| Arizona | 2.606 (0.742) | 2.327 (0.787) | 3.037 (0.691) | 2.465 (0.767) |
| California | 4.186 (0.78) | 3.315 (0.854) | 5.357 (0.747) | 4.502 (0.808) |
| Colorado | 5.003 (0.572) | 3.55 (0.563) | 5.393 (0.513) | 3.684 (0.564) |
| Idaho | 5.889 (0.748) | 6.247 (0.639) | 5.718 (0.771) | 6.563 (0.617) |
| Montana | 4.634 (0.727) | 4.276 (0.719) | 7.002 (0.722) | 5.819 (0.732) |
| Nevada | 3.154 (0.794) | 2.698 (0.816) | 3.212 (0.78) | 3.706 (0.668) |
| New Mexico | 3.089 (0.643) | 2.75 (0.606) | 3.109 (0.629) | 2.955 (0.554) |
| Oregon | 4.9 (0.681) | 4.782 (0.693) | 7.171 (0.701) | 8.509 (0.638) |
| Utah | 3.93 (0.753) | 3.364 (0.803) | 4.204 (0.702) | 3.292 (0.797) |
| Washington | 4.748 (0.658) | 7.63 (0.371) | 5.229 (0.7) | 9.246 (0.426) |
| Wyoming | 5.238 (0.529) | 8.214 (0.651) | 4.811 (0.543) | 7.696 (0.65) |
| **By Season** | | | | |
| Fall | 5.478 (0.658) | 6.346 (0.56) | 6.917 (0.69) | 8.536 (0.56) |
| Spring | 2.577 (0.737) | 2.075 (0.817) | 2.77 (0.697) | 2.136 (0.794) |
| Summer | 5.191 (0.7) | 5.242 (0.688) | 6.635 (0.708) | 6.861 (0.698) |
| Winter | 3.994 (0.832) | 3.688 (0.857) | 4.436 (0.794) | 4.061 (0.825) |

# Supplementary Table 3. RMSE values for the spatial folds results (shown in Table 3 in the main text) normalized (divided) by the mean of each corresponding observation subset.

For example, the RMSE for the 2008-2016 model in Arizona was divided by the mean of the observations in Arizona from 2008-2016. This provides a sense of the predictive performance relative to the observed values.

|  | **2008-2016 Ensemble Model Training RMSE, Normalized** | **2008-2016 Ensemble Model Testing RMSE, Normalized** | **2008-2018 Ensemble Model Training RMSE, Normalized** | **2008-2018 Ensemble Model Testing RMSE, Normalized** |
| --- | --- | --- | --- | --- |
| **By PM_2.5_ Level (µg/m^3^)** | | | | |
| Below 35 | 0.45 | 0.52 | 0.56 | 0.56 |
| Below 60 | 0.47 | 0.53 | 0.59 | 0.57 |
| Below 150 | 0.50 | 0.57 | 0.64 | 0.62 |
| Below 300 | 0.54 | 0.58 | 0.69 | 0.66 |
| Below 500 | 0.58 | 0.66 | 0.74 | 0.75 |
| Below 1000 | 0.63 | 0.68 | 0.80 | 0.80 |
| **By Year** | | | | |
| 2008 | 0.45 | 0.54 | 0.55 | 0.54 |
| 2009 | 0.45 | 0.53 | 0.55 | 0.55 |
| 2010 | 0.48 | 0.52 | 0.59 | 0.54 |
| 2011 | 0.48 | 0.51 | 0.58 | 0.53 |
| 2012 | 0.67 | 0.98 | 0.76 | 1.04 |
| 2013 | 0.58 | 0.69 | 0.68 | 0.70 |
| 2014 | 0.61 | 0.66 | 0.73 | 0.68 |
| 2015 | 0.77 | 0.67 | 0.88 | 0.71 |
| 2016 | 1.04 | 0.76 | 1.12 | 0.81 |
| 2017 | NA | NA | 1.05 | 0.99 |
| 2018 | NA | NA | 0.93 | 1.14 |
| **By State** | | | | |
| Arizona | 0.51 | 0.66 | 0.60 | 0.61 |
| California | 0.47 | 0.40 | 0.64 | 0.51 |
| Colorado | 1.10 | 0.65 | 1.62 | 0.66 |
| Idaho | 0.91 | 1.49 | 0.98 | 1.26 |
| Montana | 0.81 | 0.77 | 1.03 | 0.97 |
| Nevada | 0.53 | 0.53 | 0.63 | 0.56 |
| New Mexico | 0.70 | 1.87 | 0.71 | 2.07 |
| Oregon | 0.76 | 1.46 | 1.13 | 1.61 |
| Utah | 0.64 | 0.68 | 0.73 | 0.64 |
| Washington | 0.90 | 0.70 | 1.04 | 1.29 |
| Wyoming | 1.65 | 0.91 | 1.49 | 0.89 |
| **By Season** | | | | |
| Fall | 0.72 | 0.79 | 0.91 | 0.98 |
| Spring | 0.50 | 0.51 | 0.63 | 0.54 |
| Summer | 0.71 | 0.71 | 0.89 | 0.87 |
| Winter | 0.49 | 0.59 | 0.62 | 0.60 |

# Supplementary Table 4. RMSE values for the random folds results (shown in Table 3 in the main text) normalized (divided) by the mean of each corresponding observation subset.

For example, the RMSE for the 2008-2016 model in Arizona was divided by the mean of the observations in Arizona from 2008-2016. This provides a sense of the predictive performance relative to the observed values.

|  | **2008-2016 Ensemble Model Training RMSE, Normalized** | **2008-2016 Ensemble Model Testing RMSE, Normalized** | **2008-2018 Ensemble Model Training RMSE, Normalized** | **2008-2018 Ensemble Model Testing RMSE, Normalized** |
| --- | --- | --- | --- | --- |
| **By PM_2.5_ Level (µg/m^3^)** | | | | |
| Below 35 | 0.37 | 0.37 | 0.42 | 0.43 |
| Below 60 | 0.39 | 0.39 | 0.45 | 0.46 |
| Below 150 | 0.43 | 0.42 | 0.50 | 0.50 |
| Below 300 | 0.47 | 0.43 | 0.55 | 0.53 |
| Below 500 | 0.51 | 0.48 | 0.60 | 0.61 |
| Below 1000 | 0.56 | 0.58 | 0.67 | 0.73 |
| **By Year** | | | | |
| 2008 | 0.37 | 0.38 | 0.43 | 0.37 |
| 2009 | 0.38 | 0.33 | 0.44 | 0.34 |
| 2010 | 0.39 | 0.35 | 0.43 | 0.35 |
| 2011 | 0.41 | 0.36 | 0.44 | 0.36 |
| 2012 | 0.61 | 0.66 | 0.63 | 0.75 |
| 2013 | 0.51 | 0.45 | 0.54 | 0.45 |
| 2014 | 0.54 | 0.51 | 0.60 | 0.58 |
| 2015 | 0.69 | 0.58 | 0.74 | 0.64 |
| 2016 | 0.94 | 1.28 | 0.96 | 1.24 |
| 2017 | NA | NA | 0.92 | 1.03 |
| 2018 | NA | NA | 0.80 | 1.08 |
| **By State** | | | | |
| Arizona | 0.42 | 0.38 | 0.48 | 0.39 |
| California | 0.42 | 0.33 | 0.52 | 0.44 |
| Colorado | 0.92 | 0.66 | 0.95 | 0.65 |
| Idaho | 0.76 | 0.81 | 0.72 | 0.83 |
| Montana | 0.69 | 0.64 | 0.94 | 0.78 |
| Nevada | 0.46 | 0.39 | 0.47 | 0.54 |
| New Mexico | 0.59 | 0.53 | 0.59 | 0.56 |
| Oregon | 0.71 | 0.69 | 0.96 | 1.13 |
| Utah | 0.54 | 0.46 | 0.58 | 0.46 |
| Washington | 0.76 | 1.22 | 0.80 | 1.41 |
| Wyoming | 1.24 | 1.94 | 1.12 | 1.79 |
| **By Season** | | | | |
| Fall | 0.67 | 0.77 | 0.80 | 0.99 |
| Spring | 0.41 | 0.33 | 0.45 | 0.35 |
| Summer | 0.64 | 0.65 | 0.76 | 0.78 |
| Winter | 0.42 | 0.39 | 0.48 | 0.44 |

# Supplementary Table 5: Variable Importance metrics by model

| 2008-2016 model (includes CMAQ PM_2.5_) | | 2008-2018 model (no CMAQ PM_2.5_) | | |
| --- | --- | --- | --- | --- |
| **Variable** | **Importance** | **Variable** | **Importance** |  |
| NLCD_5km | 100.00 | Population density | 100.00 |  |
| NLCD_10km | 66.68 | Elevation | 54.63 |  |
| NLCD_1km | 12.21 | Longitude | 47.74 |  |
| Elevation | 11.19 | NLCD_10km | 43.22 |  |
| Latitude | 11.07 | NLCD_5km | 42.40 |  |
| Population density | 10.24 | Surface pressure | 38.53 |  |
| Longitude | 7.97 | CMAQ | 35.86 |  |
| Temperature at 2m | 7.86 | Latitude | 33.27 |  |
| Surface pressure | 6.40 | NLCD_1km | 28.68 |  |
| State: California | 6.21 | Temperature at 2m | 21.47 |  |
| Region: Southwest | 5.47 | State: California | 19.57 |  |
| MAIAC AOD | 4.59 | Cosine of day of year | 15.11 |  |
| Cosine of day of year | 3.81 | Region: Southwest | 10.28 |  |
| Dew point temp. at 2m | 3.16 | Date | 9.58 |  |
| Date | 2.98 | Dew point temp. at 2m | 9.46 |  |
| Cosine of month | 2.84 | Cosine of month | 8.13 |  |
| Relative humidity at 2m | 2.78 | Region: Northwest | 7.95 |  |
| Planetary boundary layer height from surface | 2.07 | Region: Southwest + study period 2008-2016 | 7.71 |  |
| Sum of arterial and collector road lengths within 1000m | 1.84 | Relative humidity at 2m | 7.31 |  |
| NDVI | 1.62 | Sum of arterial and collector road lengths within 1000m | 6.52 |  |
| Sum of arterial and collector road lengths within 500m | 1.49 | Planetary boundary layer height from surface | 6.47 |  |
| Pressure reduced to mean sea level | 1.40 | MAIAC AOD | 6.03 |  |
| Region: Southwest + study period 2008-2016 | 1.34 | NDVI | 5.80 |  |
| Year | 1.30 | Sum of arterial and collector road lengths within 500m | 5.52 |  |

Top 24 variables for each model shown. Variable importance scores are relative to the top-ranking variable.

# Supplementary Table 6: Descriptive statistics for predicted daily PM_2.5_ from the 2008-2016 ensemble model

| **Subset** | **Mean (µg/m^3^)** | **% < 0 before setting to 0 (%)** | **Q1 (µg/m^3^)** | **Median (µg/m^3^)** | **Q3 (µg/m^3^)** | **Max (µg/m^3^)** |
| --- | --- | --- | --- | --- | --- | --- |
| **Year** |  |  |  |  |  |  |
| 2008 | 10.0 | 0.19 | 5.7 | 8.4 | 12.9 | 213.7 |
| 2009 | 9.0 | 0.21 | 5.4 | 7.8 | 11.5 | 132.1 |
| 2010 | 8.6 | 0.21 | 5.1 | 7.5 | 10.9 | 150.1 |
| 2011 | 8.5 | 0.23 | 5.1 | 7.5 | 10.8 | 168.5 |
| 2012 | 8.6 | 0.24 | 5.2 | 7.6 | 10.9 | 302.1 |
| 2013 | 8.5 | 0.27 | 4.9 | 7.3 | 10.8 | 244.8 |
| 2014 | 8.3 | 0.27 | 4.8 | 7.2 | 10.6 | 215.3 |
| 2015 | 8.6 | 0.27 | 5.0 | 7.2 | 10.6 | 339.5 |
| 2016 | 8.1 | 0.28 | 4.9 | 7.1 | 10.3 | 238.8 |
| **State** |  |  |  |  |  |  |
| Arizona | 7.4 | 0.16 | 5.5 | 7.1 | 8.8 | 168.5 |
| California | 11.2 | 0.21 | 7.4 | 10.1 | 13.8 | 322.5 |
| Colorado | 6.0 | 0.28 | 3.9 | 5.7 | 7.4 | 283.8 |
| Idaho | 6.8 | 0.04 | 3.9 | 5.7 | 8.1 | 192.5 |
| Montana | 5.0 | 0.83 | 2.4 | 3.9 | 6.0 | 156.6 |
| Nevada | 8.1 | 0.49 | 6.1 | 7.9 | 9.7 | 174.5 |
| New Mexico | 5.2 | 0.11 | 3.4 | 4.8 | 6.5 | 155.3 |
| Oregon | 6.4 | 0.30 | 3.7 | 5.1 | 7.6 | 236.2 |
| Utah | 7.5 | 0.26 | 4.3 | 6.0 | 8.4 | 110.5 |
| Washington | 6.6 | 0.09 | 4.0 | 5.5 | 7.9 | 339.5 |
| Wyoming | 4.3 | 1.19 | 2.5 | 3.8 | 5.4 | 100.7 |
| **Season** |  |  |  |  |  |  |
| Fall | 8.8 | 0.14 | 5.3 | 7.7 | 11.2 | 302.1 |
| Spring | 7.3 | 0.22 | 4.6 | 6.6 | 9.1 | 142.8 |
| Summer | 9.1 | 0.34 | 5.7 | 8.0 | 11.4 | 339.5 |
| Winter | 9.7 | 0.28 | 4.9 | 8.1 | 12.8 | 160.6 |

# Supplementary Table 7: Descriptive statistics for predicted daily PM_2.5_ from the 2008-2018 ensemble model

| **Subset** | **Mean (µg/m^3^)** | **% < 0 before setting to 0 (%)** | **Q1 (µg/m^3^)** | **Median (µg/m^3^)** | **Q3 (µg/m^3^)** | **Max (µg/m^3^)** |
| --- | --- | --- | --- | --- | --- | --- |
| **Year** |  |  |  |  |  |  |
| 2008 | 10.1 | 0.22 | 5.8 | 8.6 | 12.9 | 238.8 |
| 2009 | 9.3 | 0.25 | 5.6 | 8.1 | 11.8 | 212.3 |
| 2010 | 8.5 | 0.25 | 5.2 | 7.5 | 10.8 | 100.4 |
| 2011 | 9.0 | 0.26 | 5.3 | 7.9 | 11.5 | 220.3 |
| 2012 | 8.9 | 0.24 | 5.5 | 8.0 | 11.4 | 357.5 |
| 2013 | 9.0 | 0.26 | 5.4 | 7.9 | 11.4 | 297.1 |
| 2014 | 8.6 | 0.27 | 5.1 | 7.6 | 11.0 | 238.3 |
| 2015 | 8.6 | 0.26 | 5.2 | 7.5 | 10.8 | 355.0 |
| 2016 | 8.2 | 0.29 | 5.0 | 7.4 | 10.6 | 345.4 |
| 2017 | 9.4 | 0.26 | 5.1 | 7.8 | 11.6 | 432.0 |
| 2018 | 10.0 | 0.31 | 5.4 | 8.2 | 12.1 | 355.2 |
| **State** |  |  |  |  |  |  |
| Arizona | 7.5 | 0.01 | 5.6 | 7.1 | 8.8 | 220.3 |
| California | 11.6 | 0.05 | 7.9 | 10.6 | 13.9 | 432.0 |
| Colorado | 6.0 | 0.27 | 3.9 | 5.6 | 7.4 | 267.8 |
| Idaho | 7.7 | 0.19 | 4.1 | 6.2 | 8.9 | 222.0 |
| Montana | 5.7 | 3.66 | 2.5 | 4.2 | 6.6 | 260.1 |
| Nevada | 8.1 | 0.07 | 6.0 | 7.7 | 9.5 | 189.3 |
| New Mexico | 5.4 | 0.06 | 3.4 | 4.9 | 6.6 | 209.2 |
| Oregon | 7.1 | 0.42 | 3.9 | 5.5 | 8.2 | 391.7 |
| Utah | 7.7 | 0.27 | 4.4 | 6.2 | 8.8 | 156.2 |
| Washington | 7.1 | 0.47 | 4.1 | 5.7 | 8.2 | 357.5 |
| Wyoming | 4.8 | 0.18 | 2.6 | 3.9 | 5.6 | 156.4 |
| **Season** |  |  |  |  |  |  |
| Fall | 9.4 | 0.22 | 5.7 | 8.3 | 11.8 | 432.0 |
| Spring | 7.2 | 0.27 | 4.6 | 6.6 | 9.1 | 200.4 |
| Summer | 9.9 | 0.15 | 6.1 | 8.8 | 12.2 | 355.2 |
| Winter | 9.7 | 0.42 | 5.1 | 8.2 | 12.6 | 335.2 |

#### 
